# Has Psychology Become More Positive? Trends in Language Use in Article Abstracts

**DOI:** 10.3389/fpsyg.2022.870549

**Published:** 2022-05-30

**Authors:** Naomi Baes, Henry Speagle, Nick Haslam

**Affiliations:** Melbourne School of Psychological Sciences, The University of Melbourne, Parkville, VIC, Australia

**Keywords:** character strengths, language use, positive psychology, text analysis, virtues

## Abstract

The positive psychology movement, launched near the start of the twenty-first century, aimed to shift the focus of psychology away from misery, conflict, and pathology toward happiness, human flourishing, and wellbeing. However, there have been few attempts to gauge whether psychology as a whole has become more positive in its focus. This study tested this possibility by examining a corpus of 829,701 abstracts from articles published in 875 psychology journals between 1970 and 2017. Positivity was indexed by the positive emotion dictionary using the Linguistic Inquiry and Word Count tool and a newly constructed positive character dictionary. Both indices showed a steep rise through the study period, with the positive character index's rise occurring since 2000. A Negative Emotion index also rose linearly over the study period, suggesting that the rise in positive emotion might reflect in part a general increase in affective or evaluative language use. While there appears to have been an increase in psychology's positivity, that increase is complex, non-linear, and the degree to which it can be ascribed to positive psychology remains uncertain.

## Introduction

The positive psychology movement arose at the dawn of the new millennium and set itself the ambitious goal of changing psychology's focus. Its early proponents argued that while psychology may have initially started as a neutral field, its emphasis on examining the negative aspects of human nature introduced a focus on negative over positive topics. While acknowledging the importance of studying negative phenomena, such as psychopathology, prejudice, and the roots of social conflict, pioneers of the movement argued that psychology was failing to produce enough knowledge to understand what makes life worth living (Seligman and Csikszentmihalyi, 2014). To rectify this imbalance, the positive psychology movement aimed to promote an increased focus on human strengths and virtues and began emphasizing research on topics such as gratitude, happiness, resiliency, and wellbeing.

Martin Seligman's 1998 Presidential Address to the American Psychological Association (Seligman, 1998) made the mission of positive psychology clear. He called for the establishment of positively focused journals to correct psychology's disproportionate negative focus with a new emphasis on positive emotion and character strengths - that is, psychological processes or mechanisms that define and provide ways to display virtues (Peterson and Seligman, 2004). Peterson and Seligman (2004) even compiled a handbook of character strengths and virtues as a counterpoint to the Diagnostic and Statistical Manual for Mental Disorders, Fourth Edition (American Psychiatric Association, 1994). Since its inception, positive psychology has grown rapidly as a field, developing lines of research on character, happiness, hope, human flourishing, and wellbeing in a wide variety of contexts, from clinics to schools to work organizations. To the extent that this work has been influential, the content of psychology research should have shifted in a positive direction in the past two decades.

However, whether such a broad shift has taken place has yet to be determined, and there are reasons to question it. The expansive discipline of psychology may render the influence of positive psychology limited to specific subdisciplines and thus be small in magnitude. In addition, it can be argued that psychology did not entirely neglect themes of positive human functioning before the advent of positive psychology. James (1902) was writing on “healthy mindedness” one century earlier, and Gable and Haidt (2005) document how literature on such phenomena as curiosity, forgiveness, hope, and laughter existed before positive psychology was launched. Writers in the humanistic psychology tradition, in particular, shared with the positive psychology an emphasis on positive human characteristics (Allport, 1995), the fully functioning person (Rogers, 1961), self-actualization, and the study of healthy individuals (Maslow, 1968). If positive phenomena were featuring in psychology before the rise of positive psychology, the latter's impact on the field's focus might be modest.

Research exploring these trends has found limited evidence that psychology has become more positive over time and suggested that the culture at large may have become less positive in some domains. Kesebir and Kesebir (2012) found a decrease in the use of general and specific terms related to moral virtues over the course of the twentieth century in the Google Books corpus, revealing historical trends in the cultural salience of positive moral concepts. Nevertheless, it remains to be determined whether similar historical trends are found within the discourse of academic psychology and whether such trends can be observed in relation to positive phenomena beyond moral virtues, such as positive emotions.

Several studies have examined trends in emotion categories within everyday and academic language. Using the emotion-related dictionaries of the Linguistic Inquiry and Word Count software (LIWC; Pennebaker et al., 2015), DeWall et al. (2011) found a decline in the frequency of positive emotion terms and a rise in negative emotion terms in the most popular American songs from 1980 to 2007. Focusing instead on academic psychology, but again using LIWC's emotion dictionaries, Sell and Farreras (2017) analyzed 66 introductory psychology textbooks published from the 1890s to the 1990s and found a gradual decline in the use of emotionally toned language. In contrast, Vinkers et al.'s (2015) analysis of abstracts published between 1974 and 2014 across multiple scientific fields revealed an overall rise in the usage of positive and negative words. These findings indicate that emotion categories may display different historical trajectories depending on the context in which they are examined.

Text corpus-based “culturomic” methods (Michel et al., 2011) have been used to address historical trends within psychology and cognate fields. For instance, researchers used the Google Books corpus to explore the rise and fall of psychoanalytic concepts (Haslam and Ye, 2019) and moral foundations (Wheeler et al., 2019) over time. Vylomova et al. (2019) documented the increases in the relative frequency of harm-related concepts in psychology article abstracts over the past half century. Research of this kind can track shifts in the salience of specific concepts or thematic groups of concepts within psychology, indexed by changes in the relative frequency of pre-defined word sets in massive historical text corpora. This makes it an optimal but as yet untapped method to evaluate whether and when any shifts in the overall positivity of academic psychological discourse have occurred.

This study evaluates whether academic psychology has become more positive in focus in recent decades, as indicated by the rising relative frequency of positive words in a massive corpus of journal article abstracts over the period 1970–2017. Such a trend would be consistent with the possibility that positive psychology has had a substantial impact on the field of psychology as a whole, especially if the rise begins or accelerates around the turn of the millennium. This investigation indexes positivity in the following two distinct ways: (1) The relative frequency of positive sentiment-related terms in the abstracts, using the established LIWC positive emotions dictionary and (2) the relative frequency of a new dictionary of positive character-related terms, representing a moral or ethical sense of positivity rather than the affective sense captured by the emotions index. We hypothesize that both indices of positivity (Positive Emotion and Positive Character) would increase over the period examined.

## Method

### Materials

#### Psychology Corpus

To examine positivity trends in academic psychology, we used a massive corpus of psychology abstracts, first described in Vylomova et al. (2019). The corpus comprised abstracts from journals in the field of psychology, collected from E-Research and PubMed databases, covering the period 1970–2017, and represents a substantial majority of English-language psychology articles published in this period. Abstracts were selected for study because they distill the essential contributions, findings, and ideas of a research paper (Cleveland and Cleveland, 1983). Abstracts have also been successfully used as relevant sources of text to research historical trends in scientific research (Swales and Feak, 2010; Vinkers et al., 2015).

Researchers developed the corpus in 2017 by downloading the baseline database from PubMed which contained metadata for over 25 million abstracts across the health sciences. They then used ISSN numbers for 1,095 journals tagged as “Psychology” in the SClmago database to filter PubMed for matches and extracted entries for 570,857 articles from 758 of the identified psychology journals; 439,499 (77%) contained abstracts. After removing abstracts with copyright notices, corrections, retractions, and those containing less than 85 characters, there remained 428,936 abstracts published in the period 1975–2017. Researchers then used the Crossref text mining application programming interface (API) to gather meta-data for the same set of 1,095 journals by using ISSN numbers and following Crossref links to available abstracts. This procedure yielded 740,955 abstracts from 803 Psychology tagged journals beginning in 1930, resulting in 718,512 abstracts after removing book reviews, copyright notices, editorials, and non-English abstracts. The two sets of abstracts were then merged, after which the identical duplicates were removed by matching abstracts with formatting differences found using Levenstein's distance metric. This compilation process left a merged corpus of 871,340 distinct abstracts from 875 journals. Only the 829,701 abstracts published in 1970–2017 were used in the present analysis.

The corpus was pre-processed into an analyzable format by lower-casing letters and removing punctuation. We used the Linguistic Inquiry and Word Count (LIWC; Pennebaker et al., 2015) software program to compute our variables. LIWC counts the word frequencies of pre-programmed and custom-made “dictionaries” (i.e., sets of phrases, terms, and word stems) in text corpora, and returns percentages of words in a corpus that represents each dictionary.

#### Dictionary Development

To assess affective (sentiment-related) positivity, we used the LIWC 2015 “Positive Emotion” dictionary, which contains 620 words (e.g., “happy”), word stems (e.g., “positivi^*^”), and word phrases [e.g., “(will) like”]. The asterisk in the word stem items instructs LIWC to ignore successive letters of possible words to capture all word forms based on a base form or word stem, whereby the uniquely inflected word form is treated as a single item. This dictionary has previously been shown to have the internal consistency reliabilities of 0.23 (corrected Spearman Brown's alpha) and 0.64 (uncorrected Cronbach's alpha) (Pennebaker et al., 2015). We also computed the corresponding LIWC 2015 “Negative Emotion” index, which uses a dictionary comprising 744 words (e.g., “hurt,” “nasty,” and “ugly”), and word stems (e.g., “aggravate^*^”). It demonstrates the internal consistency reliabilities of 0.55 (corrected Spearman Brown's alpha) and 0.17 (uncorrected Cronbach's alpha) (Pennebaker et al., 2015).

To assess character-related positivity, we created a custom dictionary. First, we developed a set of terms distinctive to positive character, drawn from Peterson and Seligman's (2004) handbook of character strengths and virtues. All terms were examined as nouns only (i.e., no other morphological variants such as adjectives) because character strengths are usually presented as names for virtues, and this was the focus of our positive character dictionary. The initial set of 32 one- to three-word terms contained the labels of the six proposed core virtues (e.g., “courage” and “humanity”) and their 26 component character strengths (e.g., “integrity” and “kindness”). These 32 terms were then independently judged by three authors on the following exclusion criterion: “Exclude any term used in psychology in a manner not referring to a character strength or virtue (i.e., as a positively valued individual difference variable).” Nine terms were recommended for exclusion by a majority of the judges: “beauty”, “citizenship”, “curiosity”, “fairness”, “humanity”, “innovation”, “love”, “perspective”, and “temperance”. These terms were excluded because they were judged not to be used exclusively to refer to character attributes. For example, “beauty” commonly refers to the esthetic appreciation of objects, “perspective” often refers to an intellectual standpoint rather than a kind of wisdom, “fairness” often refers to a distribution of resources rather than a quality of a fair-minded person, and “citizenship” often refers to an objective demographic fact about a person rather than a set of civic virtues. This left a final Positive Character dictionary of 23 terms as displayed in Table 1. It demonstrated a split-half reliability of 0.41, based on using Spearman Brown's correction applied to the median 0.26 correlation between randomly selected halves of the positive character terms.

**Table 1:** Positive character dictionary.

**Term**
Bravery Courage Creativity Forgiveness Gratitude Hope Humility Humo(+u)r Integrity Justice Kindness Leadership Love of learning Open-mindedness Persistence Prudence Self-control Social intelligence Spirituality Transcendence Vitality Wisdom Zest

### Measures

The LIWC Positive Emotion and Negative Emotion indices represent the summed relative frequency of the words in the respective dictionaries in each year from 1970 to 2017 (i.e., the summed count of the positive or negative word set in each year divided by the total number of words in abstracts from that year). The Positive Character index, albeit distinct from the emotion-based sentiment indices, was generated in the same way: The new custom-made dictionary of 23 terms was inputted into LIWC to compute the annual relative frequency of the positive character words as a proportion of all words in that year of the corpus. The Positive Character dictionary file for input into LIWC and the indices output for all dictionaries are publicly available at https://osf.io/v4uhr/.

## Results

**Figure 1:**
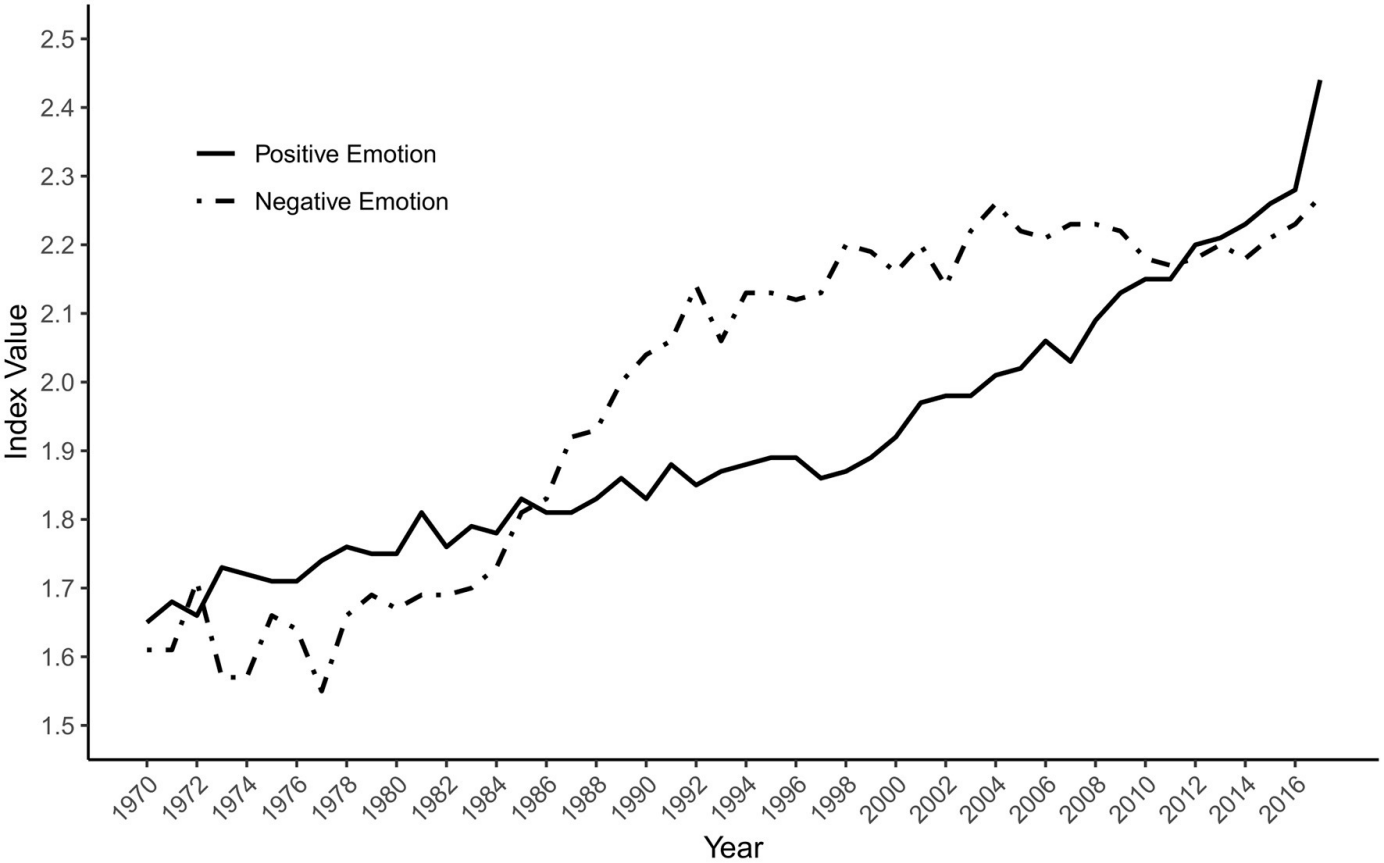
The positive emotion and negative emotion indices by year.

Figure 1 presents scores of the Positive Emotion and Negative Emotion indices for every year of the study period. The Positive Emotion index rises from 1.65 in 1970 to 2.44 in 2017, a relative increase of 48%. Scores on the index correlate 0.95, 95% CI (0.92, 0.97), with year (*p* < 0.001), supporting our hypothesis that positive sentiment will increase over the study period. However, the activity observed in the Negative Emotion index shows a similar increase, rising from 1.61 in 1970 to 2.27 in 2017, a relative increase of 41% [*r*_(46)_ = 0.93, 95% CI (0.87, 0.96), *p* < 0.001]. Therefore, rather than a unique increase for positive emotions, there appears to have been a generalized increase in emotion-related language in the abstracts over the study period. However, Figure 1 indicates that the respective time series differ in the timing of their increases. The Positive Emotion index rises steadily with a subtle acceleration since the late 1990s, whereas the Negative Emotion index rises steeply in the 1980s, although it remains relatively stable in the final two decades of the series.

**Figure 2:**
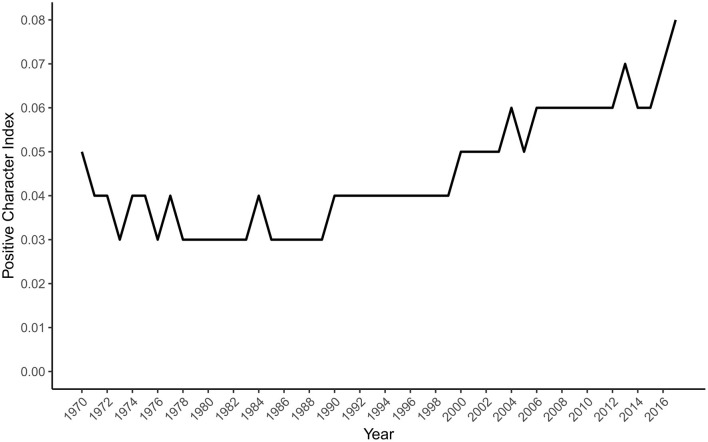
The positive character index by year.

The time series of the Positive Character index is presented in Figure 2. It shows that the positive character terms appeared infrequently but rose steeply from the late 1990s to the end of the period. The index correlated strongly with year [*r*_(46)_ = 0.82, 95% CI (0.69, 0.89), *p* < 0.001], again consistent with our hypothesis that positive character will also increase over the study period.

**Figure 3:**
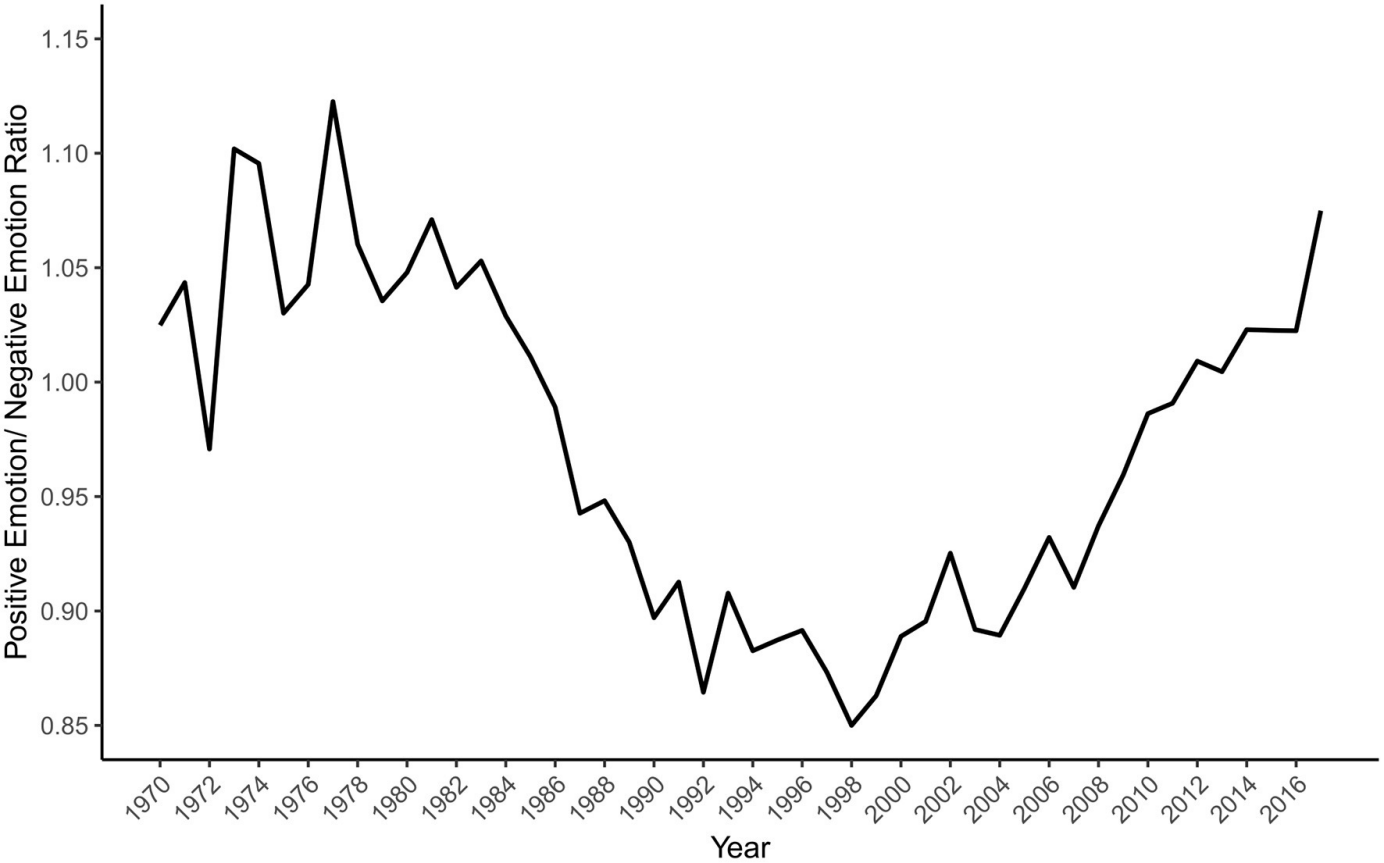
Ratio of the positive emotion and negative emotion indices by year.

These differential patterns are clarified by Figure 3 which presents the ratio of the two indices (Positive Emotion/Negative Emotion). Positive Emotion outweighs Negative Emotion from 1970 to the mid-1980s, after which the pattern reverses until the late 1990s. Subsequently, the index demonstrates a substantial upward trend, pointing to a steeper rise in positive emotion terms over negative emotion terms in the abstracts.

To validate the two positivity indices, we assessed whether they were elevated in abstracts of the positive psychology articles. Abstracts from all articles published in the prominent *Journal of Positive Psychology* (2006–2017) were extracted from the corpus, and LIWC was used to compute their Positive Emotion and Positive Character indices. As expected, the mean annual Positive Emotion index value for the positive psychology abstracts (6.96) was much higher than for the corpus as a whole (1.92) or for the corpus during the same 2006–2017 period (2.19). Likewise, the mean Positive Character index for the positive psychology abstracts (1.03) noticeably exceeded the index for the whole corpus (0.04) or for the equivalent period of the whole corpus (0.06). These findings that the LIWC Positive Emotion and Positive Character indices were more than three times and 17 times higher, respectively, in the positive psychology abstracts than in all abstracts, strongly supports their validity as indices of positivity in this study.

## Discussion

Our analysis of the abstracts of nearly one million psychology journal articles offers qualified support for our hypotheses and provides clues to why and when the rising positivity of psychology may have occurred. The Positive Emotion index was based on a massive, broad, and well-validated LIWC dictionary of positively evaluated terms, and the Positive Character index was newly constructed using character strengths and virtues that represent a moral and ethical sense of positivity. In support of our hypotheses, both indices demonstrated a substantial rise in psychology abstracts over the study period, from 1970 to 2017.

On our first hypothesis, the substantial increase in positive sentiment-related terms over the study period appears to offer strong support for our prediction that positive emotion has increased in psychology. However, that support is qualified by the almost equally substantial rise in the Negative Emotion index. One interpretation of this pattern of findings is that, in recent decades, psychology research has tended to focus on emotion and evaluation more than in earlier decades, perhaps due in part to the rise of affective science (Dukes et al., 2021). An alternative interpretation is that the pattern reflects stylistic changes in psychology writing, perhaps specific to abstracts, in which more expressive language has become acceptable, desirable, or expected. For instance, Wheeler et al. (2021) demonstrated a rise in informal writing within academic psychology abstracts. Although this second interpretation runs counter to the decreased use of emotionally toned words found in psychology textbooks by Sell and Farreras (2017), it may be consistent with the increased use of both positive and negative words in the scientific abstracts from 1970 to 2014 documented by Vinkers et al. (2015). In either case, the findings indicate that the rising emotional positivity in psychology abstracts in recent years may be more consistent with a rise in emotionality than with a distinctive rise in positivity.

The findings related to our second hypothesis, based on our new Positive Character index, are less ambiguous. The index, representing a moral and ethical sense of positivity, demonstrated a steep overall rise from 1970 to 2017. There was clear evidence that the 23 terms had increased substantially in their prevalence in more recent psychology abstracts, off a low base. This pattern is in marked contrast to the decline in the use of character terms in the Google Books corpus found by Kesebir and Kesebir (2012). By implication, the growth of positivity in psychology abstracts may be more evident when positivity is conceptualized in a relatively narrow moral or normative sense rather than it being understood as a broad semantic domain of positive evaluation and emotion.

If positive psychology has indeed played a role in the growing representation of positive concepts in psychology abstracts, that role may be stronger for character-related positivity than for emotion-related positivity. It could be argued that by using a dictionary grounded in positive psychology, we may have tipped the scales in favor of the predicted rise in positive character terms. However, as the terms are well-established English words rather than new coinages by positive psychologists, and articles that directly examine the VIA taxonomy (Peterson and Seligman, 2004) are rare, we believe this critique is not a fatal one. Although the dictionary is novel and small, compared to the LIWC emotion-related dictionaries, it has excellent content validity through its inclusion in Peterson and Seligman (2004) established account of character strengths and virtues.

The descriptive approach taken by our research does not allow us to test whether the positive psychology movement is responsible for any changes in the salience of positive emotion or character in the corpus of psychology abstracts. Rather than directly testing the causal impact of the positive psychology movement, we merely tested whether a rise in positivity, consistent with such an impact, has occurred. With these caveats in mind, we can speculate on how temporal patterns in the data are or are not compatible with positive psychology's potential influence. Two trends are consistent with that influence. First, the Positive Character index shows a steep rise beginning around 1998, around when the positive psychology movement launched. Second, the Positive Emotion index rises more steeply after 2000 than before. That trend is sharpened when the ratio of Positive to Negative Emotion is considered. Contrary to the view that psychology was biased away from positive phenomena before positive psychology's emergence, a clear rise in relative positivity starts in 1999, albeit one that returns the ratio to levels that prevailed in the 1970s. It is uncertain how much the earlier peak of positivity reflected the influence of humanistic psychologists, and how much the relative lack of focus on negative emotion before the growth of clinical psychology as a robust profession and an academic field of study into the 1980s (Benjamin, 2005). When considering that academic psychology is mostly neutral (Gable and Haidt, 2005), it is plausible that specific movements within psychology have influenced fluctuations in its affective temperature.

### Study Limitations

The present research has some limitations. First, although the abstracts provide dense and standardized summaries of psychological research, they may not be the ideal medium for assessing the overall positivity or focus of that research. Abstracts combine neutral reporting on concepts, findings, and methods with an advertising function, and their stylistic aspects, and requirements may distort the assessment of their content focus (Wheeler et al., 2021). A more comprehensive assessment might employ full article texts, although this is prohibitively difficult due to copyright restrictions. Furthermore, the Positive Emotion and Negative Emotion dictionaries could be partitioned into their components (e.g., disappointment and happiness) to represent the various dimensions present in emotion. Second, our analysis combined abstracts published in a particular year into a single text for the purposes of analysis, and future research might explore changes in language use at the individual abstract level (e.g., examining proportions of abstracts containing positive language)—taking care to compute precision and recall estimates. Third, the new Positive Character dictionary is small and has yet to be thoroughly validated; therefore, the findings based on it must be regarded as provisional. An expanded dictionary that is not directly based on positive psychology models of the structure of virtues and character strengths might also be preferable, potentially combining work from other researchers (e.g., Buckingham and Clifton, 2001) and stoic philosophers (Sherman, 2021), and parts of speech other than nouns might also be included. Fourth, our analyses do not allow us to answer causal questions about the extent to which positive psychology is responsible for changes in the positivity of psychology as a whole. Such questions are challenging to address, not least because it is difficult to isolate a new field of study as a causal influence in big data analyses while controlling for other possible factors. For instance, it is difficult to control for the linguistic positivity bias, which holds that people tend to use positive language more than negative language (Kloumann et al., 2012; Iliev et al., 2016), and the negativity bias purportedly instilled by clinical psychology's focus on disorder and disease (Seligman and Csikszentmihalyi, 2014). Future research may begin to investigate whether particular movements have influenced trends revealed by the positivity indices used in this study by replicating their patterns in clinical and positive psychology journals, and journals representing other areas of psychology and comparing their trajectories. Articles within the corpus might be grouped by relevant journals, providing the opportunity for a text-by-text analysis of indices within areas of psychology (e.g., analysis of article abstracts within positive psychology journals to represent the positive psychology movement). Finally, although word count-based approaches to studying change in text corpora are popular and often revealing, it is important to acknowledge that word counts fail to capture the importance of context in word usage (Enfield, 2014). Future research might employ machine learning techniques that take linguistic context into account (e.g., where the positivity indices contain thorough information about the context of word occurrences, including hedging or negation; Enríquez et al., 2016).

## Conclusion

In conclusion, our study offers the first evidence that the discourse of academic psychology, at least as represented in the abstracts of journal articles, has become, in some respects, more positive over the past 50 years. When positivity is understood in a moral sense, as virtue and character, there has been an upsurge in attention within the literature beginning around the turn of the last century. When positivity is understood in a broader and more affective sense, there has been a more gradual rise that is similar in magnitude to a rise in negative emotion. Although this pattern may represent a general rise in attention to emotion and evaluation within psychology, rather than a specifically positive shift, there is some evidence of a subtle swing toward positivity at around the time when positive psychology was founded. Whether positive psychology can be credited for these subtle changes in the field of psychology as a whole remains to be determined.

## Data Availability Statement

Publicly available datasets were analyzed in this study. This data can be found at: https://osf.io/5nzse/.

## Author Contributions

HS, NB, and NH contributed to conceptualizing, designing, analyzing, and writing up the research. NB handled data curation, formal analysis, and data visualization. NH handled supervision and funding acquisition. NB and NH wrote the original draft and handled project administration. All authors contributed to the article and approved the submitted version.

## Funding

The research reported in this manuscript was supported by Australian Research Council Discovery Project DP210103984.

## Conflict of Interest

The authors declare that the research was conducted in the absence of any commercial or financial relationships that could be construed as a potential conflict of interest.

## Publisher's Note

All claims expressed in this article are solely those of the authors and do not necessarily represent those of their affiliated organizations, or those of the publisher, the editors and the reviewers. Any product that may be evaluated in this article, or claim that may be made by its manufacturer, is not guaranteed or endorsed by the publisher.

## References

[B1] Allport G. W. (1995). Becoming: Basic Considerations for a Psychology of Personality. Vol. 20. New Haven, Conn: Yale University Press.

[B2] American Psychiatric Association (1994). Diagnostic and Statistical Manual of Mental Disorders. 4th edn. Washington, DC: American Psychiatric Association.

[B3] Benjamin L. T. Jr. (2005). A history of clinical psychology as a profession in America (and a glimpse at its future). Annu. Rev. Clin. Psychol. 1, 1–30. 10.1146/annurev.clinpsy.1.102803.143758 17716080

[B4] Buckingham M. Clifton D. O. (2001). Now, Discover Your Strengths. New York: Free Press.

[B5] Cleveland D. B. Cleveland A. D. (1983). Introduction to Indexing and Abstracting. Littleton, CO: Libraries Unlimited Inc.

[B6] DeWall C. N. Pond R. S. Jr. Campbell W. K. Twenge J. M. (2011). Tuning in to psychological change: linguistic markers of psychological traits and emotions over time in popular US song lyrics. Psychol. Aesthet. Creativity Arts 5, 200. 10.1037/a0023195

[B7] Dukes D. Abrams K. Adolphs R. Ahmed M. E. Beatty A. Berridge K. C.. (2021). The rise of affectivism. Nat. Hum. Behav. 5, 816–20. 10.1038/s41562-021-01130-8 34112980 PMC8319089

[B8] Enfield N. J. (2014). The Utility of Meaning: What Words Mean and Why. Oxford: Oxford University Press. 10.1093/acprof:oso/9780198709831.001.0001

[B9] Enríquez F. Troyano J. A. López-Solaz T. (2016). An approach to the use of word embeddings in an opinion classification task. Expert Syst. Appl. 66, 1–6. 10.1016/j.eswa.2016.09.005

[B10] Gable S. L. Haidt J. (2005). What (and why) is positive psychology? Rev. Gen. Psychol. 9, 103–110. 10.1037/1089-2680.9.2.103

[B11] Haslam N. Ye L. (2019). Freudian slip? The changing cultural fortunes of psychoanalytic concepts. Front. Psychol. 10, 1489. 10.3389/fpsyg.2019.01489 31316440 PMC6611072

[B12] Iliev R. Hoover J. Dehghani M. Axelrod R. (2016). Linguistic positivity in historical texts reflects dynamic environmental and psychological factors. Proc. Natl. Acad. Sci. U.S.A. 113, E7871–E7879. 10.1073/pnas.1612058113 27872286 PMC5150390

[B13] James W. (1902). The Varieties of Religious Experience: A Study in Human Nature. Longmans, Green and Co. 10.1037/10004-000

[B14] Kesebir P. Kesebir S. (2012). The cultural salience of moral character and virtue declined in twentieth century America. J. Positive Psychol. 7, 471–480. 10.1080/17439760.2012.715182

[B15] Kloumann I. M. Danforth C. M. Harris K. D. Bliss C. A. Dodds P. S. (2012). Positivity of the English language. PLoS ONE 7, e29484. 10.1371/journal.pone.0029484 22247779 PMC3256157

[B16] Maslow A. H. (1968). Toward a Psychology of Being. New York, NY: Van Nostrand Reinhold.

[B17] Michel J. B. Shen Y. K. Aiden A. P. Veres A. Gray M. K. Google Books Team. (2011). Quantitative analysis of culture using millions of digitized books. Science 331, 176–182. 10.1126/science.1199644 21163965 PMC3279742

[B18] Pennebaker J. W. Boyd R. L. Jordan K. Blackburn K. (2015). The Development and Psychometric Properties of LIWC2015. Austin, TX: University of Texas at Austin.

[B19] Peterson C. Seligman M. E. (2004). Character Strengths and Virtues: A Handbook and Classification. Vol. 1. New York, NY: Oxford University Press.

[B20] Rogers C. R. (1961). On Becoming a Person: A Therapist's View of Psychotherapy. Boston, MA: Houghton-Mifflin.

[B21] Seligman M. E. Csikszentmihalyi M. (2014). “Positive psychology: an introduction,” in M. Csikszentmihalyi, editor. *Flow and the Foundations of Positive Psychology: The Collected Works of Mihaly Csikszentmihalyi*. (Dordrecht: Springer), 279–298. 10.1007/978-94-017-9088-8_18

[B22] Seligman M. E. P. (1998). The President's address. APA. Am. Psychol. 54, 559–562.

[B23] Sell J. Farreras I. G. (2017). LIWC-ing at a century of introductory college textbooks: have the sentiments changed?. Proc. Comput. Sci. 118, 108–112. 10.1016/j.procs.2017.11.151

[B24] Sherman N. (2021). Stoic Wisdom: Ancient Lessons for Modern Resilience. New York, NY: Oxford University Press. 10.1093/oso/9780197501832.001.0001

[B25] Swales J. M. Feak C. B. (2010). “From text to task: putting research on abstracts to work,” in M. F. Ruiz-Garrido, J. C. Palmer-Silveira, and I. Fortanet-Gomez, editors. *English for Professional and Academic Purposes*. (Amsterdam: Rodopi). 169–182. 10.1163/9789042029569_012

[B26] Vinkers C. H. Tijdink J. K. Otte W. M. (2015). Use of positive and negative words in scientific PubMed abstracts between 1974 and 2014: retrospective analysis. BMJ 351, h6467. 10.1136/bmj.h6467 26668206 PMC4677695

[B27] Vylomova E. Murphy S. Haslam N. (2019). “Evaluation of semantic change of harm-related concepts in psychology,” in Proceedings of the 1st International Workshop on Computational Approaches to Historical Language Change. (Florence: Association for Computational Linguistics), 29–34. 10.18653/v1/W19-4704

[B28] Wheeler M. A. McGrath M. J. Haslam N. (2019). Twentieth century morality: the rise and fall of moral concepts from 1900 to 2007. PLoS ONE 14, e0212267. 10.1371/journal.pone.0212267 30811461 PMC6392263

[B29] Wheeler M. A. Vylomova E. McGrath M. J. Haslam N. (2021). More confident, less formal: stylistic changes in academic psychology writing from 1970 to 2016. Scientometrics 126, 9603–9612. 10.1007/s11192-021-04166-9

